# Comparative Analysis of Live and Heat-Killed *Lacticaseibacillus paracasei* K56 in Alleviating Functional Constipation

**DOI:** 10.3390/nu18182991

**Published:** 2026-09-12

**Authors:** Zhi Zhao, Xinyi Li, Jian He, Ran Wang, Lianhang Lan, Jingjing He, Jie Guo, Yixuan Li, Wen Zhao, Xiaoxia Li

**Affiliations:** 1 Department of Nutrition and Health, Key Laboratory of Functional Dairy, Co-Constructed by Ministry of Education and Beijing Government, China Agricultural University, Beijing 100190, China; 2 College of Food Science and Engineering, Gansu Agricultural University, Lanzhou 730070, China; 3 National Center of Technology Innovation for Dairy, Hohhot 100118, China

**Keywords:** *Lacticaseibacillus paracasei* K56, functional constipation, postbiotics, fecal metabolites, gut microbiota, loperamide, heat-killed bacteria

## Abstract

Background: Functional constipation is a common gastrointestinal disorder that impairs quality of life. Although probiotics have shown benefits in constipation, whether live and heat-killed forms of the same strain act through similar mechanisms remains unclear. This study compared the effects of live and heat-killed *Lacticaseibacillus paracasei* K56 in experimental constipation. Methods: Loperamide-treated mice received live or heat-killed *L. paracasei* K56. Gastrointestinal motility, colonic histology, neurotransmitters, aquaporin expression, gut microbiota, short-chain fatty acids, and metabolomic profiles were evaluated. Results: Both preparations relieved constipation, as reflected by shorter defecation latency, increased fecal output and moisture, and faster intestinal transit, with live K56 producing greater improvement. Colonic injury was alleviated, accompanied by reduced Aqp2 and Aqp3 expression and normalization of 5-hydroxytryptamine, substance P, and vasoactive intestinal peptide. The treatments increased *Lactobacillus* and *Lachnospiraceae*, promoted butyrate production, and altered tryptophan metabolism. Heat-killed K56 was mainly associated with changes in phenylalanine/tyrosine- and methionine-related metabolism, whereas live K56 showed more pronounced alterations in arginine/polyamine, tryptophan, and bile acid metabolism. Conclusions: Live and heat-killed *L. paracasei* K56 improved constipation through shared and distinct mechanisms involving gut microbiota, microbial metabolism, enteric neurotransmission, and intestinal water homeostasis. Live K56 produced stronger effects, whereas heat-killed K56 retained substantial biological activity.

## 1. Introduction

Functional constipation (FC) is a common and debilitating gastrointestinal disorder mainly marked by infrequent bowel movements, hard or dry stools, and ongoing straining or an uncomfortable feeling of incomplete evacuation [1]. Based on the Rome IV criteria, there is an epidemiological consensus that FC affects over 14% of the global population, with its prevalence increasing due to demographic aging and shifts in modern lifestyles [2,3]. Beyond severely compromising quality of life, chronic intractable constipation escalates the risks of severe colorectal [2], cardiovascular [4], and neurodegenerative pathologies [5]. Currently, FC management relies heavily on osmotic and stimulant laxatives; however, prolonged administration frequently induces adverse sequelae—including abdominal distress, drug dependency, and resistance [6]. Consequently, the development of safer and more effective intervention strategies has become a research hotspot in the field of functional constipation.

In recent years, probiotics have emerged as a promising alternative owing to their capacity to maintain microbial homeostasis, fortify the intestinal barrier, and modulate neuro-immune axes [7,8]. Crucially, the therapeutic efficacy of probiotics in mitigating FC is highly strain-specific, operating through diverse physiological pathways. Accumulating evidence indicates that specific strains can alleviate experimental constipation by unlocking distinct mechanisms, such as upgrading carbohydrate utilization via unique gene clusters (e.g., abfA) [9], stimulating colonic mucin secretion to promote lubrication [10], or restoring intestinal muscularis thickness to enhance contractile responses [11]. However, a deepening of probiotic research reveals that these health benefits do not exclusively rely on cellular viability, but are largely driven by metabolites, cell lysates, and structural components produced during fermentation [12]. These factors have been formally termed “postbiotics” by the International Scientific Association for Probiotics and Prebiotics (ISAPP) [13]. Distinct from viable strains, postbiotics circumvent the necessity for active gastrointestinal colonization or survival, thereby conferring superior biosafety, technological stability, and prolonged shelf-life during industrial processing [14]. Although recent clinical evidence confirms that postbiotic interventions can significantly increase defecation frequency and mitigate abdominal discomfort [15], the precise molecular, microbial, and metabolic mechanisms underlying these postbiotic-mediated laxative effects remain poorly understood.

*Lacticaseibacillus paracasei* K56 is a safe, fermentative commercial strain isolated from the gut of healthy children, commonly used in the development of fermented dairy products. Its live form has been confirmed to regulate gut microbiota, enhance barrier function, and modulate immunity [16,17,18]. However, research regarding K56 intervention in constipation remains scarce, particularly the lack of systematic comparison between the mechanisms of its live and heat-killed forms (postbiotics). Consequently, this investigation uses a loperamide-induced mouse model of constipation to systematically assess the impact of live and heat-killed K56 on FC relief. We examined how they influenced defecation parameters, tissue structure, and the concentrations of inflammatory cytokines and neurotransmitters in both serum and tissue. Furthermore, through non-targeted metabolomics and gut microbiota structure analysis, we explored the mechanisms by which live K56 and heat-killed K56 (postbiotics) ameliorate constipation, laying the groundwork for creating probiotic solutions to alleviate constipation.

## 2. Materials and Methods

### 2.1. Strain Culture and Heat-Killed Sample Preparation

*L. paracasei* K56 was provided by the Inner Mongolia Dairy Technology Research Center (Hohhot, China). The strain was incubated at 37 °C in MRS broth, which was commercially sourced from Beijing Land Bridge Technology Co., Ltd., Beijing, China. *L. paracasei* K56 was cultured overnight, centrifuged at 12,000× *g* for 10 min, washed thrice with PBS, and resuspended to reach a concentration of 1 × 10^9^ CFU/mL. The concentration was determined using both spectrophotometry and the plate count method to prepare the live *L. paracasei* K56 suspension (1 × 10^9^ CFU/mL), corresponding to an OD600 of 1.5. Heat-killed cells at 1 × 10^9^ cell-equivalent/mL were prepared by inactivating PBS-resuspended bacteria at 121 °C for 10 min. Both live and heat-killed K56 samples were prepared fresh daily.

### 2.2. Design and Experimental Animals

The Experimental Animal Ethics Committee of PONY Testing International Group Co., Ltd. (Beijing, China) (No. PONY-2023-FL-25; approved on 23 December 2023) approved this study, which was carried out following the ‘Guidelines for Ethical Review of Laboratory Animal Welfare’ (GB/T 35892-2018). A total of sixty 6-week-old female BALB/c mice, with body weights ranging from 18 to 22 g, were acquired from Beijing Spelford Biotechnology Co., Ltd. (Beijing, China). The mice were kept in an environment with a temperature of 22 ± 2 °C, humidity of 45 ± 10%, and a 12 h cycle of light and darkness.

After a one-week acclimatization period, the mice were randomly assigned to the experimental groups using a random allocation procedure, with 10 mice in each group. Control group, Loperamide (Xian Janssen Pharmaceutical Ltd., Xi’an, China) model group (Model), Polyethylene Glycol 4000 powder (Beaufour Ipsen Industrie, Dreux, France) positive control group (PEG 4000), Live K56 group (1 × 10^8^ CFU/d) (K56-L), and Heat-killed K56 group (1 × 10^8^ celle-quivalent/d) (K56-D). The duration of the experiment was 31 days, with 10 days dedicated to modeling and 21 days to treatment. Throughout the experiment, mice in the model and treatment groups received loperamide hydrochloride by oral gavage at 15 mg/kg body weight once daily to induce constipation. During the intervention period (days 11-31), specific treatments were administered: the PEG 4000 group was treated with 0.1 mL of a solution containing Polyethylene Glycol 4000 at 0.17 g/kg body weight, in contrast to the other groups, which received 0.1 mL of live or heat-killed K56. Following the experiment, animals were euthanized, and their serum and colon tissues were collected under sterile conditions and stored at −80 °C.

### 2.3. Measurement of Fecal Parameters

Fecal parameters were measured as briefly described by Zhang Q et al. [19]. After a 12 h fast, 0.2 milliliters of activated charcoal suspension was administered to the mice via gavage. The defecation latency (defined as the time elapsed until the first expulsion of charcoal-indexed black stool) and the cumulative number of fecal pellets discharged within a 6 h window was recorded. To determine the fecal water content (FWC), freshly voided feces were dried to a constant mass at 105 °C for 24 h. The FWC value was mathematically derived according to the following gravimetric formula:

FWC (%) = [(Wet weight − Dry weight)/Wet weight] × 100%

### 2.4. Determination of Intestinal Motility

To evaluate intestinal transit dynamics, a 0.1 mL volume of an indicator suspension (comprising 0.6% carmine and 10% Gum Arabic) was administered via gavage to mice subjected to a 12 h fast. At a precise interval of 30 min following administration, the whole bowel tract was isolated from the euthanized rodents. The propulsive velocity was subsequently derived from the ratio between the leading travel distance of the red dye (S2) and the total anatomized intestinal length (S1), adhering to the following formula:

D (small intestinal propulsion rate) = S2/S1 × 100%

### 2.5. Histological Analysis of the Colon

Approximately 0.6 cm of tissue from the distal colon was collected from a site approximately 3 cm proximal to the anal verge, fixed in 4% paraformaldehyde for 24 h, dehydrated, embedded, and then sectioned. Sections were stained with H&E and AB-PAS for histopathological observation. Images were processed using CaseViewer 2.8. (3DHISTECH Ltd., Budapest, Hungary).

### 2.6. Biochemical Analysis of Serum and Colon Tissue

To evaluate neurochemical and barrier-associated alterations, the expression profiles of vasoactive intestinal peptide (VIP), substance P (SP), 5-hydroxytryptamine (5-HT), and aquaporins (Aqp2 and Aqp3) within blood serum and localized tissues were determined. These protein levels were verified using specific Enzyme-Linked ImmunoSorbent Assay (ELISA) formulations obtained from Shanghai Enzyme-linked Biotechnology (Shanghai, China), adhering strictly to the operational workflows authorized by the supplier.

### 2.7. Microbiota Analysis of Colon Contents

#### 2.7.1. DNA Extraction and 16S rRNA Gene Sequencing

Total genomic DNA from murine colonic digesta was extracted using the solid-phase extraction protocol of the HiPure Soil DNA Kit (Magen, Guangzhou, China) according to the manufacturer’s instructions. DNA integrity was assessed by 1% agarose gel electrophoresis, and DNA concentration and purity were determined using a NanoDrop 2000 spectrophotometer (Thermo Fisher Scientific (China) Co., Ltd., Shanghai, China). The V3-V4 region of the bacterial 16S rRNA gene was amplified using the core primers 338F (5′–ACTCCTACGGGAGGCAGCAG–3′) and 806R (5′–GGACTACHVGGGTWTCTAAT–3′). Each 20 μL PCR reaction contained 4 μL of 5× TransStart FastPfu buffer, 2 μL of 2.5 mM dNTPs, 0.8 μL of each primer (5 μM), 0.4 μL of TransStart FastPfu DNA polymerase, and 10 ng of template DNA. PCR amplification was performed at 95 °C for 3 min, followed by 27 cycles of 95 °C for 30 s, 55 °C for 30 s, and 72 °C for 30 s, with a final extension at 72 °C for 10 min.

The amplified products were purified using a PCR Clean-Up Kit (Shanghai Meiji Yuhua Biomedical Technology Co., Ltd., Shanghai, China) following 2% agarose gel electrophoresis and quantified using a Qubit 4.0 fluorometer (Thermo Fisher Scientific (China) Co., Ltd., Shanghai, China). Sequencing libraries were prepared using the NEXTFLEX Rapid DNA-Seq Kit and sequenced on an Illumina NextSeq 2000 platform (Majorbio Bio-Pharm Technology Co., Ltd., Shanghai, China).

#### 2.7.2. Sequence Processing and Taxonomic Assignment

Raw paired-end reads were processed using fastp v0.23.4 for quality control and FLASH v1.2.11 for sequence merging. During quality filtering, bases with a quality score below 20 were trimmed using a 50 bp sliding window, reads shorter than 50 bp after filtering were discarded, and reads containing more than five ambiguous bases were removed. Paired-end reads were merged with a minimum overlap of 10 bp and a maximum mismatch ratio of 0.2 in the overlapping region. Sample-specific barcodes and primers were used for demultiplexing, with no barcode mismatches and up to two primer mismatches allowed. The quality-filtered and merged sequences were denoised using the DADA2 plugin in QIIME2, and the resulting features were defined as amplicon sequence variants (ASVs). Taxonomic classification was performed using the classify-sklearn Naive Bayes classifier in QIIME2 against the SILVA 138 16S bacterial database, with a confidence threshold of 70%. Chloroplast and mitochondrial sequences were removed before downstream analyses. To reduce the influence of differences in sequencing depth, the feature table was rarefied to 30,355 sequences per sample across all 30 samples. After quality control and filtering, a total of 9473 ASVs were obtained.

### 2.8. Quantification of Short-Chain Fatty Acids (SCFAs)

#### 2.8.1. Reference Formulation and Matrix Spiking

Ethanoic, propanoic, and butanoic fractions were mixed into a standard solution and dissolved in diethyl ether, sourced from Sigma-Aldrich, Germany, and of analytical grade. A 2-ethylbutanoic acid isomer served as the internal reference marker, with a fixed volume inoculated into all specimen vials before downstream processing.

#### 2.8.2. Gas Chromatography Coupled with Mass Spectrometry (GC-MS)

Analytes underwent processing using gas-phase separation setups coupled to flame-ionization detection systems. Structural isolation was achieved over DB-FFAP capillary columns (0.32 mm × 30 mm × 0.5 μm), utilizing a thermal gradient tracking program initiated at 80 °C and terminating at 220 °C. Target short-chain fatty species were detected via distinct temporal elution positions and calculated using derived linear validation curves.

### 2.9. Fecal Metabolomics Analysis

#### 2.9.1. Sample Preparation

Weighed at 50 mg, the lyophilized feces were homogenized in 1 mL of an extraction solution made of methanol and water in a 4:1 volume ratio, containing an internal standard of 2 μg/mL. After thorough shaking, the samples were subjected to ultrasonic extraction at 4 °C for 30 min and then stored at −20 °C for an hour to aid in protein precipitation.

#### 2.9.2. Ultra-Performance Liquid Chromatography-Tandem Mass Spectrum Profiling

The samples were analyzed using an ultra-performance liquid chromatography system (LC-40D X3, Shimadzu (Shanghai) Global Laboratory Consumables Co., Ltd., Shanghai, China) coupled to a quadrupole time-of-flight mass spectrometer (TripleTOF 7600, AB SCIEX, Marlborough, MA, USA). In positive ion mode, chromatographic separation was performed on a BEH C8 column (2.1 mm × 100 mm, 1.7 μm) with a C8 guard column (2.1 mm × 10 mm, 1.7 μm; Waters, Milford, MA, USA). The column temperature was maintained at 50 °C. The mobile phases were water containing 0.1% formic acid (A) and acetonitrile containing 0.1% formic acid (B). The gradient was set as follows: 10% B from 0 to 1 min, 10–40% B from 1 to 5 min, 40–100% B from 5 to 17 min, and 100% B from 17 to 22 min. B was then reduced from 100% to 10% within 0.1 min, followed by a 2.9 min equilibration period.

For negative ion mode, an HSS T3 column (2.1 mm × 100 mm, 1.8 μm) with a T3 guard column (2.1 mm × 10 mm, 1.8 μm; Waters, Milford, MA, USA) was used. The column was also maintained at 50 °C. Mobile phase A was 6.5 mM ammonium bicarbonate (NH_4_HCO_3_) in water, and mobile phase B was 6.5 mM NH_4_HCO_3_ in methanol/water (95:5, *v*/*v*). The gradient was as follows: 0% B from 0 to 1 min, 0–40% B from 1 to 3 min, and 40–100% B from 3 to 16 min, followed by 100% B from 16 to 22 min. B was decreased to 0% within 0.1 min and maintained at 0% for 2.9 min for equilibration. The flow rate was 0.35 mL/min, and 5 μL of each sample was injected.

Mass spectrometric data were acquired in information-dependent acquisition (IDA) mode. The ion source gas 1 and gas 2 were set at 50 and 55 psi, respectively, with a curtain gas of 30 psi and a source temperature of 500 °C. The declustering potential was set at 60 V in positive ion mode and −60 V in negative ion mode, while the ion spray voltage was 5500 V and −4500 V, respectively. TOF-MS data were collected over an *m*/*z* range of 50–1200 with an accumulation time of 150 ms, and dynamic background subtraction was enabled. Product-ion scans were acquired over an *m*/*z* range of 30–1200 with an accumulation time of 50 ms. The collision energy was set at 35 V and −35 V for positive and negative ion modes, respectively, with a collision energy spread of 15 V. Zeno pulsing was enabled.

Raw LC-MS data were processed using MS-DIAL version 5.0 Peak detection, compound annotation, and subsequent metabolomics data processing were performed using MS-DIAL. Metabolites were annotated by matching MS and MS/MS information against the Human Metabolome Database (HMDB) and METLIN databases. For data preprocessing, variables with non-zero values in at least 80% of the samples in at least one experimental group were retained. Missing values were replaced by the minimum value in the original data matrix. To minimize variation associated with sample preparation and instrumental instability, peak intensities were normalized using the sum normalization method. Variables with a QC relative standard deviation (RSD) greater than 30% were removed. The resulting data matrix was then log10 transformed for subsequent analyses.

### 2.10. qPCR Analysis of Colon Gene Expression

#### 2.10.1. RNA Extraction and cDNA Synthesis

Total RNA were harvested from colonic mucosal segments using an optimized phenol-chloroform liquid extraction vehicle (TRIzol). First-strand complementary DNA (cDNA) libraries were subsequently generated utilizing a 1 μg fraction of the isolated template mass, executing the transcription step via PrimeScript reverse-transcriptase reagent blocks (Takara Biomedical Technology (Beijing) Co., Ltd., Beijing, China).

#### 2.10.2. Real-Time Kinetic Gene Amplification Scaling

Quantitative real-time PCR (qRT-PCR) was performed using a LightCycler 480 instrument with SYBR Green fluorescent dye. Custom oligonucleotides matching structural genes (AQP2 [20], AQP3 [21], IL-6, TNF-α [22]) along with the reference gene GAPDH were synthesized targeting Mus musculus genomic alignments. Using the 2^−ΔΔCt^ method, the expression levels of target genes were normalized against GAPDH.

### 2.11. Statistical Analysis

The data are presented as the mean plus or minus the standard deviation (SD). Differences among groups were analyzed using one-way analysis of variance (ANOVA), followed by Tukey’s multiple comparisons test. Using GraphPad Prism version 9.5, statistical analyses were carried out, considering a *p*-value < 0.05 as statistically significant.

## 3. Results

### 3.1. K56 and Its Postbiotics Improve Defecation Parameters in Constipated Mice

**Figure 1:**
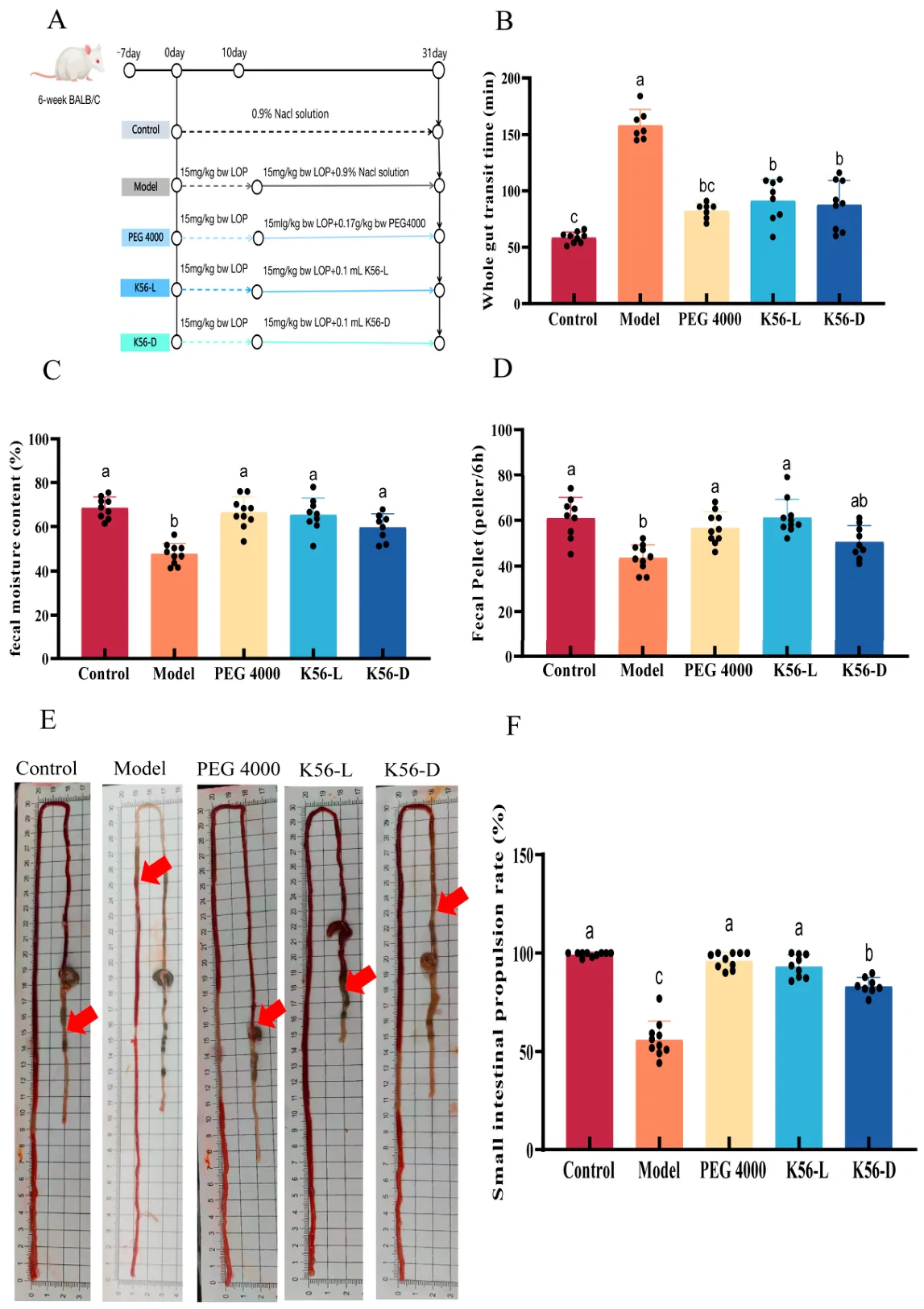
Therapeutic effects of live K56 and postbiotics on loperamide-induced constipated mice. (**A**) Schematic of experimental design. (**B**) Whole gut transit time (*n* = 10 mice/group). (**C**) Fecal water content (%). (**D**) 6 h fecal pellet count. (**E**,**F**) Intestinal transit rate (arrows indicate carmine migration distance). Mean values with different letters over the bars are significantly different (*p* < 0.05) according to an ordinary one-way ANOVA test.

To evaluate the therapeutic efficacy of K56 and its postbiotic form against functional constipation, mice were challenged with loperamide and subsequently administered either live K56 (K56-L) at 10^8^ CFU/day or heat-killed K56 (K56-D) at 10^8^ cell-equivalent/d for three weeks (Figure 1A). Efficacy was assessed by measuring whole gut transit time, 6 h fecal pellet count, fecal water content, and small intestinal propulsion rate. Results indicated that Model treatment significantly prolonged whole gut transit time relative to the Control group (Control: 58.67 ± 4.93 min vs. Model: 158.30 ± 13.85 min, *p* < 0.01). The interventions with K56-L (91.50 ± 6.55 min) and K56-D (88.00 ± 7.09 min) significantly decreased the time to first defecation compared to the Model group, approaching the results of the PEG 4000 control group (82.43 ± 6.90 min, *p* < 0.01) (Figure 1B). Evaluating changes in constipation can be done by examining FWC (Figure 1C). FWC was significantly reduced in the Model group relative to the Control group (Control: 68.63 ± 4.82% vs. Model: 47.70 ± 4.71%, *p* < 0.01). Both K56-L (65.45 ± 7.51%) and K56-D (59.70 ± 6.07%) interventions significantly restored the Model-induced reduction in moisture content (*p* < 0.01). Data for the 6 h fecal pellet count are shown in Figure 1D; the Control group had higher levels than the significantly reduced Model group (Control: 61.00 ± 9.00 vs. Model: 43.50 ± 5.72, *p* < 0.01). In contrast, only the K56-L intervention (61.30 ± 7.79) significantly improved the fecal pellet count (*p* < 0.01). The small intestinal propulsion rate represents intestinal transit efficiency (Figure 1E,F). Compared to the Model group (56.36 ± 9.10%), both K56-L and K56-D interventions significantly increased the intestinal propulsion rate (*p* < 0.01). At the same time, the improvement rate in the K56-L group (91.52 ± 5.67%) was significantly higher than that in the K56-D group (83.45 ± 4.18%) (*p* < 0.05). These results demonstrate that both K56-L and K56-D can enhance intestinal motility and effectively alleviate constipation symptoms in mice.

### 3.2. K56 and Its Postbiotics Ameliorate Colonic Tissue Damage in Constipated Mice

**Figure 2:**
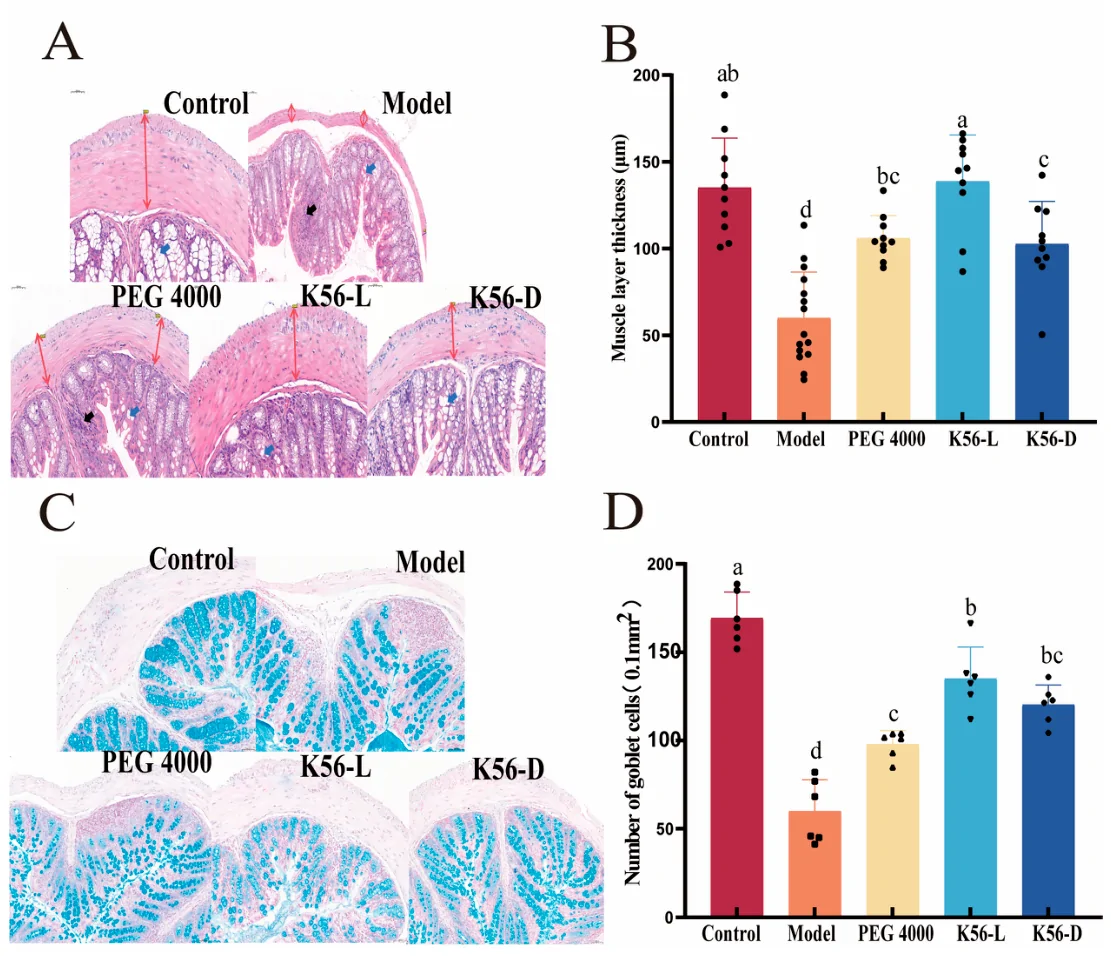
Colonic tissue morphology stained with Hematoxylin and Eosin (H&E). (**A**) H&E staining results of colon tissue. (**B**) Thickness of colonic muscle layer. (**C**) AB-PAS staining results of colon tissue. (**D**) Number of goblet cells per 0.1 mm^2^ in mouse colonic tissue. Red double-headed arrows indicate muscle layer thickness, black arrows indicate inflammatory cell infiltration, and blue arrows indicate goblet cells. Mean values with different letters over the bars are significantly different (*p* < 0.05) according to an ordinary one-way ANOVA test. Scale bar = 0.050 mm (A).

After three weeks of treatment with K56 and its postbiotics, changes in the distal colon tissue structure of constipated mice are depicted in Figure 2A. The distal colon sections stained with H&E in the Control group exhibited preserved epithelial morphology, featuring organized mucosal and smooth muscle layers (Figure 2A-Control). In line with other studies, the colon tissue of mice treated with Model exhibited decreased crypt depth, thinning of both mucosal and muscle layers, and infiltration of inflammatory cells into the damaged mucosa (Figure 2A-Model). These results indicate that Model treatment specifically induced colonic inflammation and structural damage. Conversely, treatment with live K56 (K56-L) and heat-killed K56 (K56-D) significantly increased muscle layer thickness (*p* < 0.05) and reduced inflammatory cell infiltration, thereby reversing histological alterations and restoring the mucosal integrity compromised by loperamide (Figure 2A,B). Mucins, produced by goblet cells, are crucial elements of the luminal mucus and act as the initial barrier against physical and chemical harm from intestinal contents [23]. The Alcian Blue stain showed that the K56-L and K56-D groups had a significantly greater number of goblet cells and crypts compared to the Model group (*p* < 0.05) (Figure 2C,D). These findings further showed that K56-L was significantly more effective than K56-D in restoring colonic muscular layer thickness (*p* < 0.05), while K56-L also showed a greater tendency to restore goblet cell numbers. The results suggest that the continuous administration of live K56 or its postbiotic components over a period of three weeks can alleviate constipation by repairing LOP-induced damage to the colon’s structure and function.

### 3.3. K56 and Its Postbiotics Improve Intestinal Dysfunction in Constipated Mice

**Figure 3:**
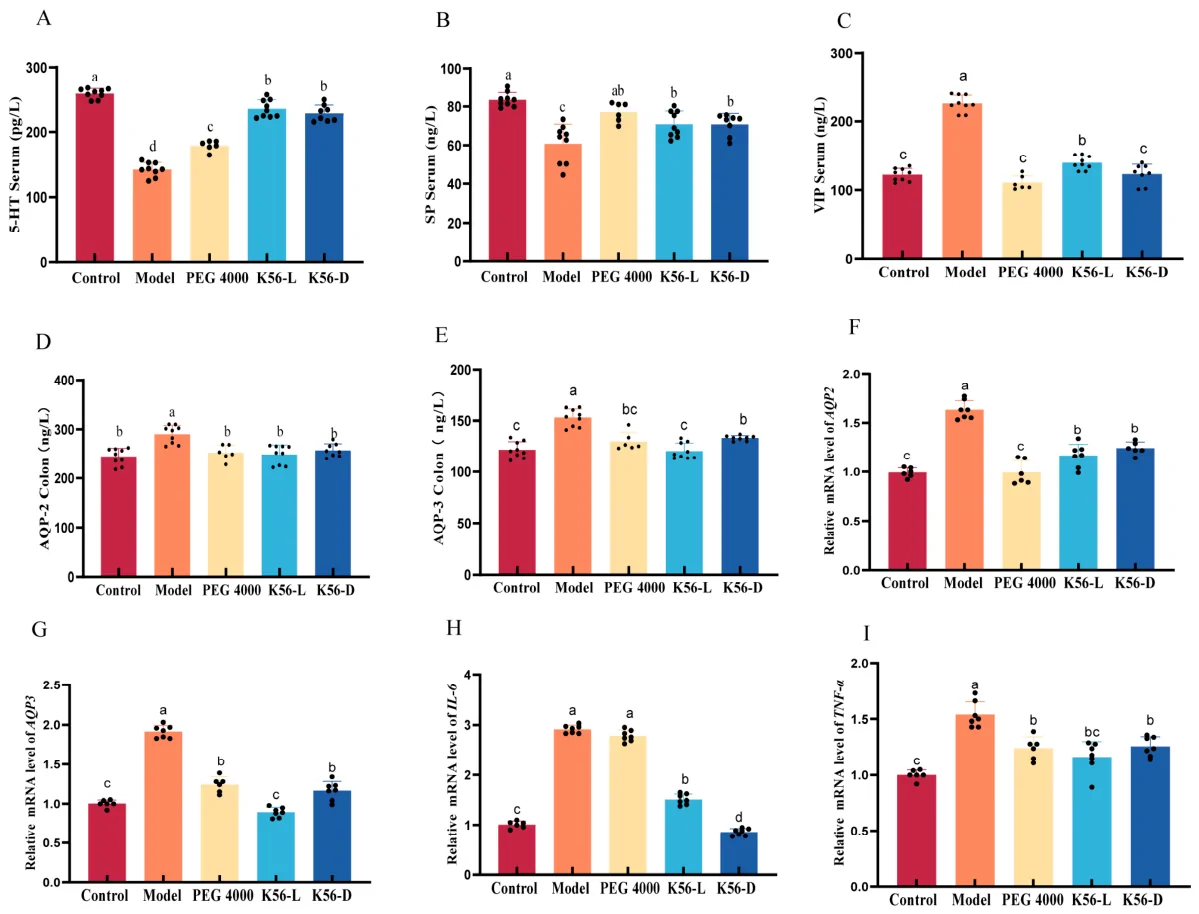
Ameliorative effects of live K56 and its postbiotics on intestinal dysfunction and inflammation. (**A**–**C**) ELISA detection of serum 5-HT, SP, and VIP levels. (**D**,**E**) ELISA detection of colonic AQP2 and AQP3 content. (**F**,**G**) RT-qPCR detection of AQP2 and AQP3 transcriptional expression in colon tissue. (**H**,**I**) RT-qPCR detection of IL-6 and TNF-α transcriptional expression in colon tissue. Mean values with different letters over the bars are significantly different (*p* < 0.05) according to an ordinary one-way ANOVA test.

We investigated the effects of K56 and its postbiotics on intestinal neurotransmitters, water-electrolyte metabolism, and inflammation in constipated mice using RT-qPCR and ELISA. As shown in Figure 3A,B, serum levels of 5-HT and the excitatory neurotransmitter SP were significantly decreased in constipated mice (*p* < 0.05 vs. Control), whereas treatment with K56-L and its postbiotics increased these levels (*p* < 0.05 vs. Model). Furthermore, as illustrated in Figure 3C–G, loperamide significantly upregulated the levels of VIP (*p* < 0.05 vs. Control) and AQPs, while K56-L and K56-D significantly reduced VIP and AQP content (*p* < 0.05 vs. Model). Concurrently, K56-L and K56-D statistically inhibited the occurrence of inflammation in constipated mice, including the mRNA levels of TNF-α and IL-6 (Figure 3H,I) (*p* < 0.05 vs. Model). Meanwhile, K56-L produced a significantly greater reduction in AQP3 expression and levels in colonic tissue than K56-D. The findings suggest that K56 and its postbiotics relieve constipation caused by loperamide by modulating intestinal neurotransmitters, enhancing water-electrolyte balance, and reducing intestinal inflammation.

### 3.4. K56 and Its Postbiotics Reshape Gut Microbiota Structure in Constipated Mice

**Figure 4:**
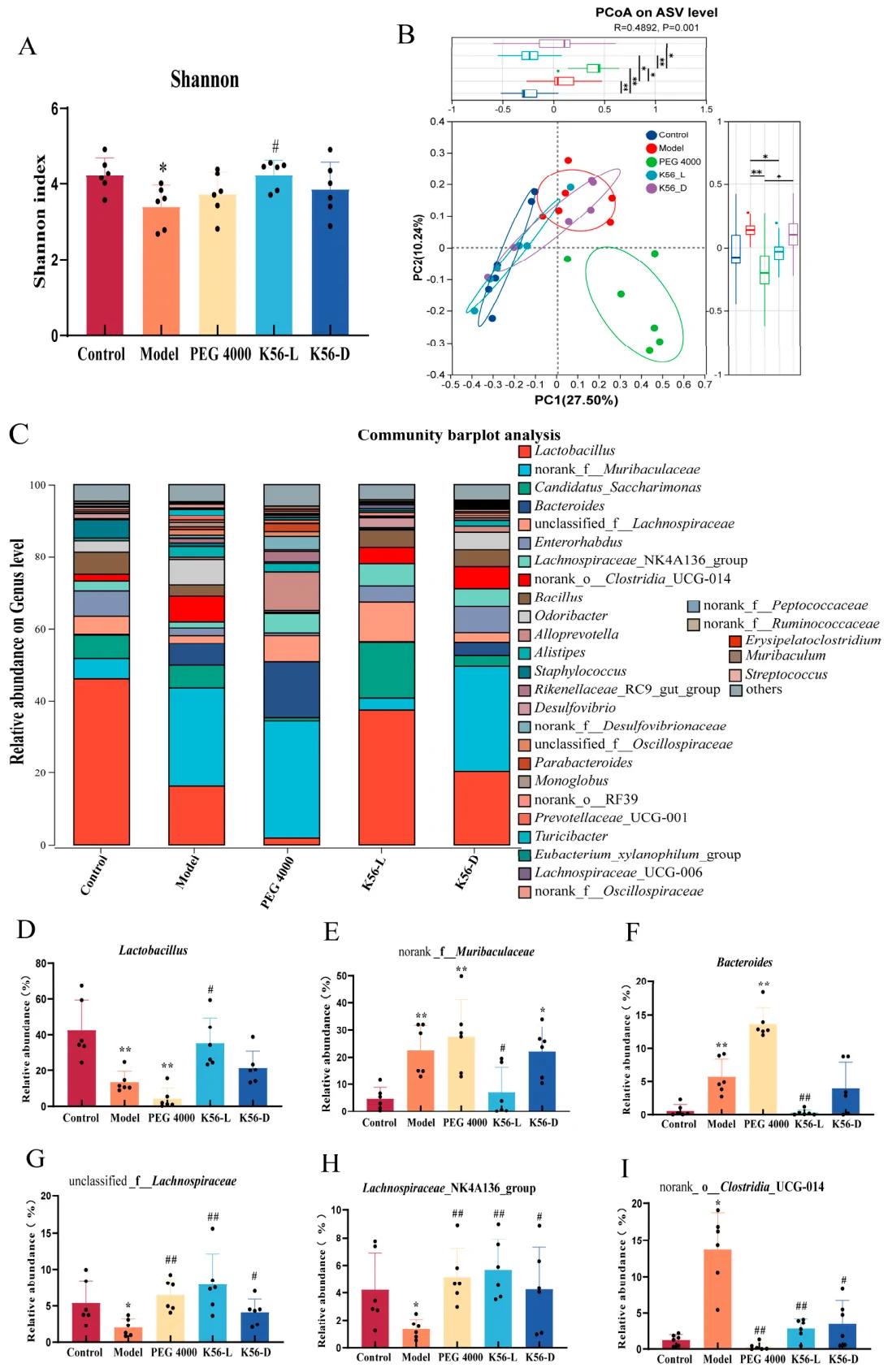
K56 and its postbiotics modulate the gut microbiota composition in constipated mice. (**A**) α-diversity analysis (Shannon index). (**B**) PCoA based on Bray-Curtis distance showing β-diversity. (**C**) Relative abundance at the genus level. (**D**) The relative abundances of the *Lactobacillus*. (**E**) The relative abundances of the norank_f__*Muribaculaceae*. (**F**) The relative abundances of the *Bacteroides*. (**G**) The relative abundances of the unclassified_f__*Lachnospiraceae*. (**H**) The relative abundances of the *Lachnospiraceae*_NK4A136_group. (**I**) The relative abundances of the norank_o__*Clostridia*_UCG-014. Data are presented as mean ± SD. * *p* < 0.05 vs. Control group; ** *p* < 0.05 vs. Control group; # *p* < 0.05 vs. Model group; ## *p* < 0.01 vs. Model group, as determined by one-way analysis of variance (ANOVA).

Recognizing the role of gut dysbiosis in causing constipation, we utilized 16S rDNA sequencing to investigate how K56-L and its postbiotic elements influence the diversity, richness, and composition of gut microbiota. A total of 4,173,296 high-quality sequencing reads (single-end) were obtained from 30 colon content samples. To reduce the influence of differences in sequencing depth, the feature table was rarefied to 30,355 sequences per sample across all 30 samples. After quality control and filtering, a total of 9473 ASVs were obtained. The Shannon index (Figure 4A) was used to estimate species diversity (α-diversity). Loperamide-induced constipation led to a significant decrease in colonic species diversity (*p* < 0.05, Model vs. Control). However, treatment with K56-L and K56-D restored diversity, with K56-L showing more significant effects (*p* < 0.05, K56-L vs. Model). The Chao1 index (Supplementary Figure S6) was used to estimate species abundance (α-diversity). Loperamide-induced constipation led to a significant decrease in colonic species richness (*p* < 0.05, model group vs. control group). However, species richness was restored following treatment with K56-L and K56-D. It also indicates that K56-L is more effective than K56-D at improving the abundance and diversity of the gut microbiota. The diversity between microbiome samples (β-diversity) was evaluated using Principal Coordinate Analysis (PCoA); the five clusters showed a certain degree of separation, reflecting different bacterial structures among the groups. PERMANOVA further supported a significant difference in microbial community composition among groups (R = 0.4892, *p* = 0.001). Following intervention, the microbiota structure in K56-L and K56-D groups tended to shift toward that of the Control group (Figure 4B).

In the Model group, there was a decrease in *Firmicutes* and *Actinobacteriota* and an increase in *Bacteroidota* at the phylum level, which was notably reversed by K56-L and postbiotic treatments (Supplementary Figure S1A). At the family level, K56-L and postbiotic interventions restored the abundance of *Lactobacillaceae* and *Lachnospiraceae* and reduced *Muribaculaceae*, *Bacteroidaceae*, and *Rikenellaceae* in constipated mice (Supplementary Figure S1B). The top five species in terms of relative abundance at the genus level included *Lactobacillus*, norank_f__*Muribaculaceae*, *Candidatus*_*Saccharimonas*, unclassified_f__*Lachnospiraceae*, and *Bacteroides* (Figure 4C). To further explore the regulatory effects of K56-L and postbiotics on colonic microbiota composition, differential species analysis was conducted at the genus level. T-tests revealed significant differences between Control, Model, K56-L, and K56-D groups (Figure 4D–I). The K56-L group significantly mitigated the reduction in abundance of *Lactobacillus*, *Lachnospiraceae*_NK4A136_group, and unclassified_f*__Lachnospiraceae* (*p* < 0.01 vs. Model), as well as the increase in norank_o__*Clostridia*_UCG-014, *Bacteroides*, and norank_f__*Muribaculaceae* (*p* < 0.05 vs. Model). The K56-D group notably boosted the levels of *unclassified*_*f*__*Lachnospiraceae* and *Lachnospiraceae*_NK4A136_group (*p* < 0.05 vs. Model). Notably, K56-L exerted a significantly stronger effect than K56-D on the abundances of *Lactobacillus*, norank_f__*Muribaculaceae*, *Bacteroides*, and unclassified_f__*Lachnospiraceae*.

### 3.5. Determination of Metabolites in Mouse Feces

**Figure 5:**
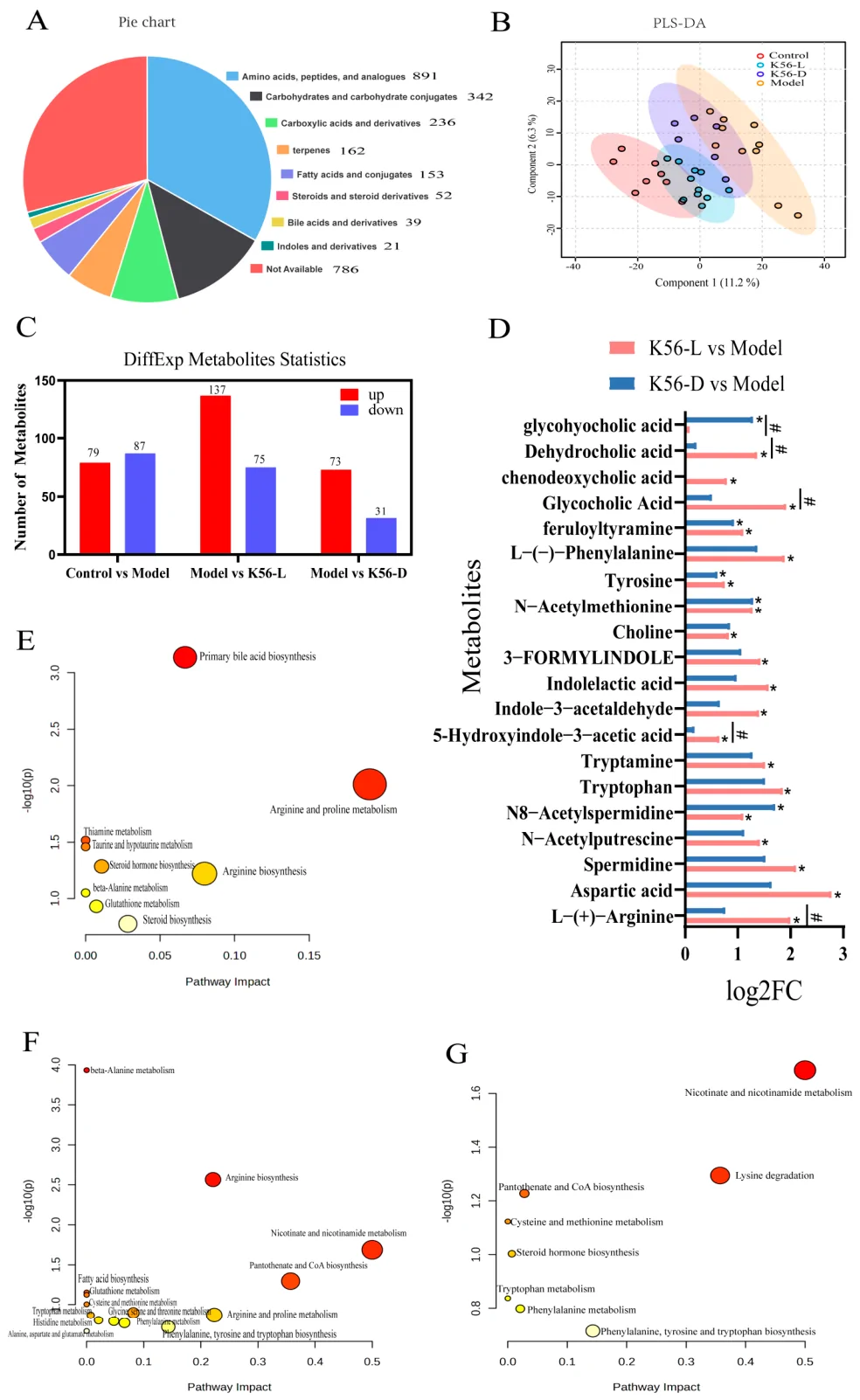
Live K56 and its postbiotics improve colonic metabolite profiles in constipated mice. (**A**) Pie chart of colonic metabolite composition. (**B**) PLS-DA plot of all samples. (**C**) Classification of differential metabolites between groups. (**D**) Log_2-_fold changes in 31 key metabolites between K56-L or K56-D and the control group. Pink: K56-L vs. control group; blue: K56-D vs. control group. (**E**–**G**) Bubble charts of pathway enrichment analysis for differential metabolites in Control vs. Model, Model vs. K56-L, and Model vs. K56-D groups. Data are presented as mean ± SD. * *p* < 0.05 vs. Model group; # *p* < 0.05: K56-L vs. K56-D group, as determined by one-way analysis of variance (ANOVA).

The study utilized UPLC-QTOF/MS to analyze fecal metabolites, aiming to further understand the role of K56-L and postbiotics in improving constipation. A total of 2682 metabolites were identified, classified as amino acids and peptides (891), carbohydrates and conjugates (342), carboxylic acids (236), terpenoids (162), fatty acids (153), steroids (52), bile acids (39), indoles (21), and other compounds (786) (Figure 5A). To identify potential biomarkers and examine different metabolic pathways, multivariate statistical analyses like Partial Least Squares Discrimination Analysis (PLS-DA) and Orthogonal Partial Least Squares Discriminant Analysis (OPLS-DA) were conducted.

The PLS-DA score plot (Figure 5B) illustrated overall differences among the four groups (Control, Model, K56-L, and K56-D). The fecal metabolite composition of the K56-L and K56-D groups was closer to the Control, suggesting that live and heat-killed K56 improved the metabolic shifts induced by loperamide. To better distinguish metabolites between groups, an OPLS-DA model was established to maximize metabolic profile differences (R2 = 0.986, Q2 = 0.880; R2 = 0.991, Q2 = 0.743; R2 = 0.997, Q2 = 0.830) (Supplementary Figure S3A–C). The model showed valid fit and no overfitting (Supplementary Figure S4A–C). The OPLS-DA model provided Variable Importance in Projection (VIP scores), and metabolites with VIP scores exceeding 1 and *p*-values below 0.05 were selected as potential biomarkers. Following K56-L intervention, 137 metabolites were significantly downregulated and 75 upregulated (*p* < 0.05); in the K56-D group, 73 metabolites were significantly downregulated and 31 increased (*p* < 0.05). K56-L induced changes in a greater number of metabolites than K56-D. 

As shown in Figure 5D, K56-L and K56-D altered the levels of several key fecal metabolites in constipated mice compared with the Model group. However, the two preparations differed in both the composition and magnitude of the metabolic changes. Compared with the Model group, K56-L treatment significantly increased the levels of several amino acids and their derivatives, including L-(+)-Arginine (log2FC = 2.01, *p* = 0.007), aspartic acid (2.78, *p* = 0.040), spermidine (2.11, *p* = 0.010), *N*-acetylputrescine (1.43, *p* = 0.032), and N8-acetylspermidine (1.11, *p* = 0.024). K56-L also significantly altered several tryptophan related metabolites, with increased levels of tryptophan (1.86, *p* = 0.025), tryptamine (1.53, *p* = 0.042), 5-hydroxyindole−3-acetic acid (5-HIAA; 0.66, *p* = 0.012), and indolelactic acid (1.59, *p* = 0.025). In addition, the bile acid-related metabolites glycocholic acid (1.92, *p* = 0.008) and dehydrocholic acid (1.37, *p* = 0.002), as well as choline (0.84, *p* = 0.001), were significantly increased following K56-L treatment. In comparison, the metabolic changes observed after K56-D treatment were more limited. Relative to the Model group, K56-D significantly increased the levels of N8-acetylspermidine (log2FC = 1.72, *p* = 0.008), *N*-acetylmethionine (1.30, *p* = 0.035), glycohyocholic acid (1.29, *p* = 0.007), tyrosine (0.62, *p* = 0.004), L-(−)-phenylalanine (1.31, *p* = 0.045), and feruloyltyramine (0.93, *p* = 0.028).

Direct comparison between K56-L and K56-D further revealed significant differences in several metabolites. Glycocholic acid (*p* = 0.022), dehydrocholic acid (*p* = 0.017), and 5-HIAA (*p* = 0.041) were significantly higher in the K56-L group than in the K56-D group. L-(+)-Arginine also differed significantly between the two groups (*p* = 0.044). In contrast, glycohyocholic acid was significantly higher in the K56-D group than in the K56-L group (*p* = 0.047). N8-acetylspermidine also tended to be higher in the K56-D group, although this difference did not reach statistical significance. In summary, live K56 and postbiotic components partially restored the metabolic levels altered by constipation.

Pathway enrichment analysis of differential metabolites between the Control and Model groups identified several significantly enriched pathways, including Primary bile acid biosynthesis, Arginine and proline metabolism, Arginine biosynthesis, beta-Alanine metabolism, Glutathione metabolism, Steroid biosynthesis, Steroid hormone biosynthesis, Thiamine metabolism, and Taurine and hypotaurine metabolism (Figure 5E). For the Model vs. K56-L comparison, the enriched metabolic pathways included beta-Alanine metabolism, Arginine biosynthesis, Nicotinate and nicotinamide metabolism, Pantothenate and CoA biosynthesis, Arginine and proline metabolism, Phenylalanine, tyrosine and tryptophan biosynthesis, Glycine, serine and threonine metabolism, Cysteine and methionine metabolism, Glutathione metabolism, and other related pathways (Figure 5F). Enriched pathways for Model vs. K56-D (Figure 5G) included Steroid hormone biosynthesis, Cysteine and methionine metabolism, and Phenylalanine, tyrosine and tryptophan biosynthesis. Following K56-L and postbiotic administration, the phenylalanine, tyrosine and tryptophan biosynthesis pathways and alanine, aspartate and glutamate metabolism pathways showed the most significant changes; tryptophan metabolism and cysteine and methionine metabolism were also upregulated, while fatty acid biosynthesis and lysine degradation were significantly downregulated. This indicates that live K56 and postbiotics can significantly alter metabolic pathways in FC mice.

### 3.6. Determination of Short-Chain Fatty Acids in Mouse Feces

**Figure 6:**
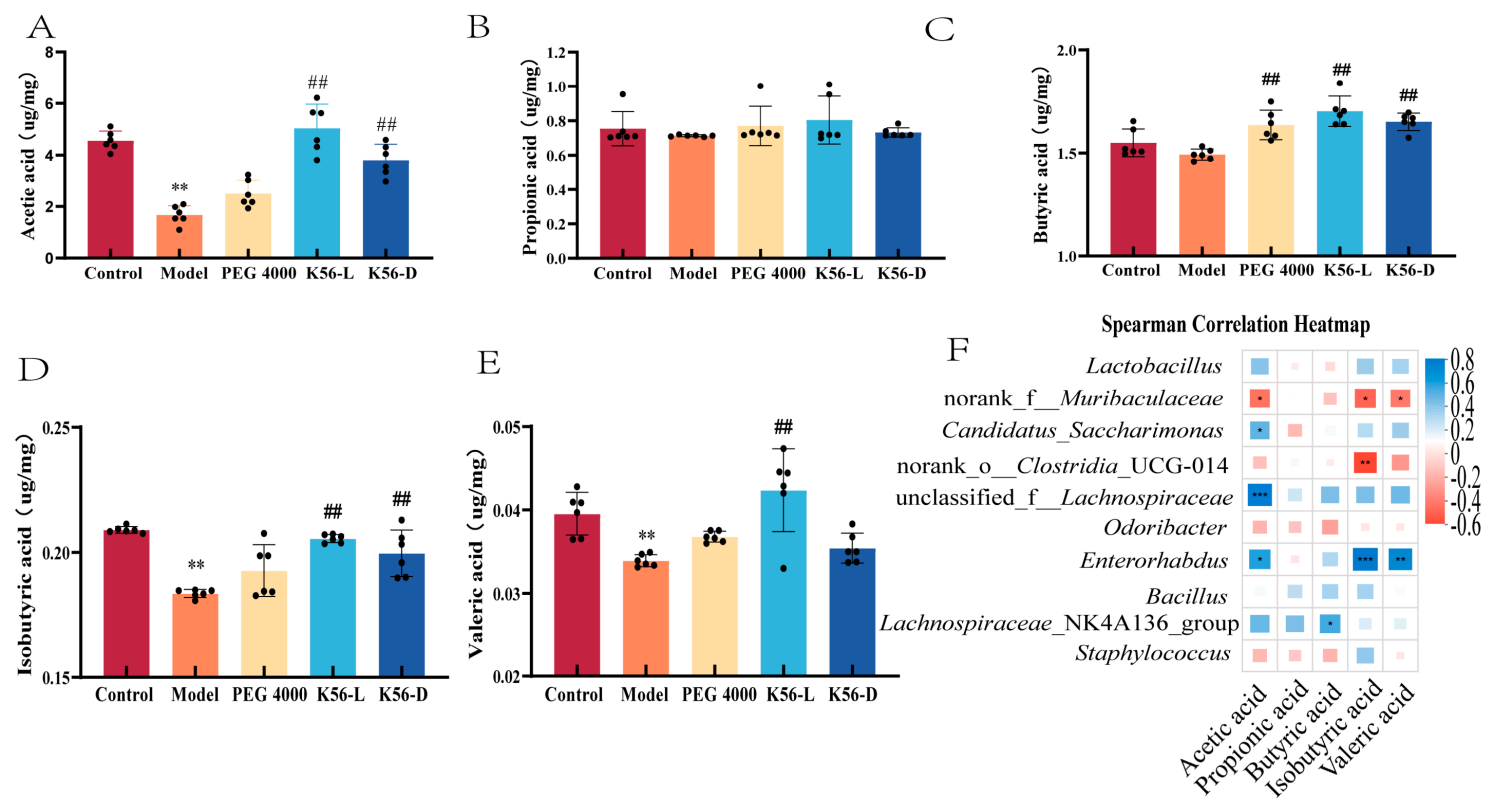
Fecal short-chain fatty acid content. (**A**) Acetic acid. (**B**) Propionic acid. (**C**) Butyric acid. (**D**) Isobutyric acid. (**E**) Valeric acid. (**F**) Heatmap of correlations between gut microbiota at the genus level and SCFAs. Data are presented as mean ± SD. * *p* < 0.05; ** *p* < 0.01 vs. Control group; *** *p* < 0.001; ## *p* < 0.01 vs. Model group, as determined by one-way analysis of variance (ANOVA).

SCFAs are a key mechanism by which probiotics alleviate chronic constipation. To determine the effect of live K56 and postbiotics on SCFA content, fecal SCFAs were quantified by GC-FID. As shown in Figure 6A–E, levels of butyric acid, acetic acid, isobutyric acid, and valeric acid in constipated mice were significantly lower than in the Control group (*p* < 0.01), while propionic acid showed a decreasing trend. K56-L intervention significantly increased the levels of all SCFAs except propionic acid (vs. Model, *p* < 0.01). K56-D intervention significantly increased butyric acid, isobutyric acid, and acetic acid (vs. Model, *p* < 0.01), while valeric acid showed alleviation without statistical difference, and propionic acid remained unchanged. Compared with K56-D, K56-L significantly increased acetic acid and valeric acid levels, while also producing greater increases in butyric acid and isobutyric acid levels.

Pearson correlation analysis was used to explore the connection between SCFA concentrations and important bacterial genera. As shown in Figure 6F, the abundances of *Lactobacillus*, unclassified_f__*Lachnospiracea*e, *Lachnospiraceae*_NK4A136_group, *Enterorhabdus*, and *Bacillus* were positively correlated with SCFA content, suggesting their potential role in SCFA synthesis. Notably, unclassified_f__*Lachnospiraceae* was significantly positively correlated with acetate (*p* < 0.001), *Enterorhabdus* with acetate, isobutyrate, and valerate (*p* < 0.05), and *Lachnospiraceae*_NK4A136_group with butyrate (*p* < 0.05). In contrast, abundances of norank_o__*Clostridia*_UCG-014, norank_f__*Muribaculaceae*, *Staphylococcus*, and *Odoribacter* were negatively correlated with SCFA content. Specifically, norank_o__*Clostridia*_UCG-014 and norank_f__*Muribaculaceae* showed significant negative correlations with most SCFA components (*p* < 0.05).

### 3.7. Correlation Analysis

**Figure 7:**
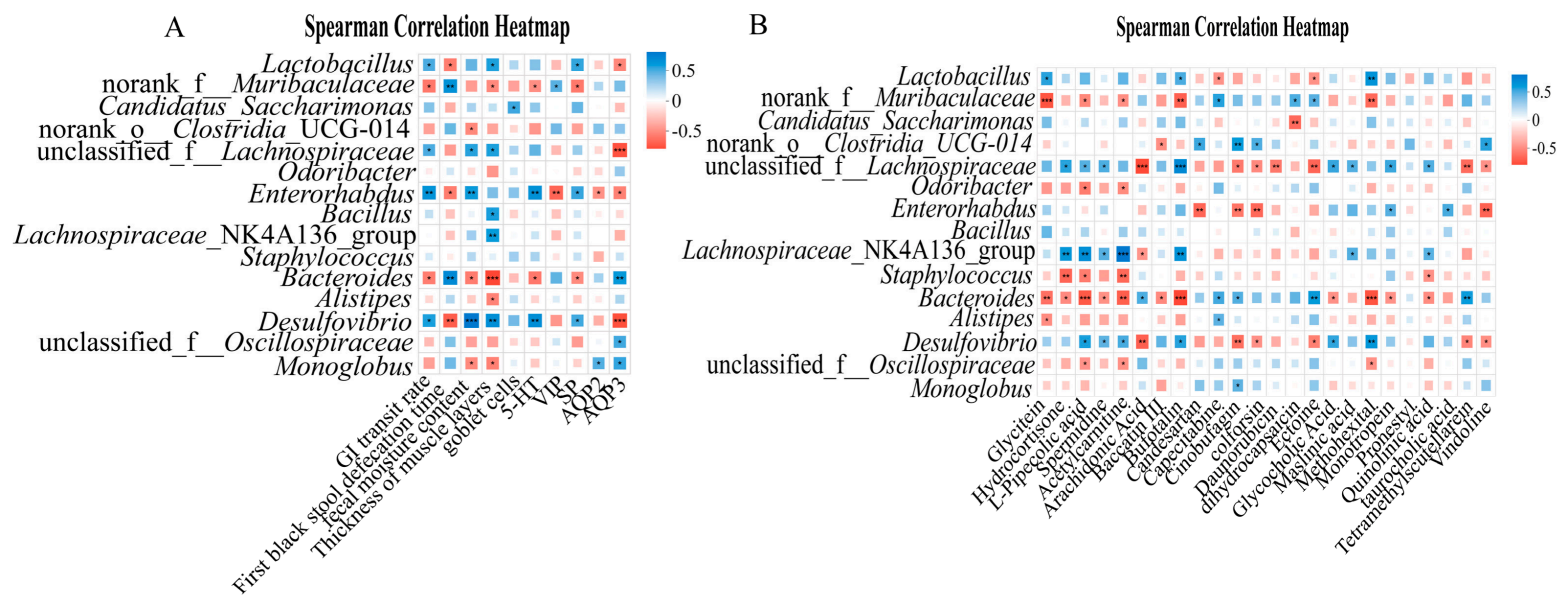
Heatmap of correlations between gut microbiota at the genus level and (**A**) constipation biomarkers and (**B**) colonic fecal metabolites. * *p* < 0.05; ** *p* < 0.01; *** *p* < 0.001, as determined by one-way analysis of variance (ANOVA).

We analyzed the relationship between genus-level gut microbiota and constipation biomarkers using Spearman correlation coefficients (Figure 7A). *Lachnospiraceae*_NK4A136_group, *Lactobacillus*, unclassified_f__*Lachnospiraceae*, *Enterorhabdus*, *Desulfovibrio*, and *Alistipes* were positively correlated with gastrointestinal motility indices (GI transit rate), goblet cell count, and 5-HT and excitatory neurotransmitters (SP), while being negatively correlated with defecation indices (first black stool time), water metabolism (AQP2, AQP3), and inhibitory neurotransmitters (VIP). Conversely, norank_o__*Clostridi*a_UCG-014, *Bacteroides*, and norank_f__*Muribaculaceae*, exhibited inverse correlations: they were positively correlated with water metabolism markers (AQP2, AQP3) and negatively correlated with GI motility and SP. Additionally, *Monoglobus* was positively correlated with water metabolism markers and negatively with GI transit rate.

Further correlation analysis was performed between colonic differential metabolites and genus-level gut microbiota (Figure 7B). The study found that most metabolites were closely related to bacteria such as *Lachnospiraceae*-related genera and *Bacteroides*. These metabolites were primarily concentrated in pathways including tryptophan metabolism, cysteine and methionine metabolism, steroid biosynthesis, and fatty acid biosynthesis. Some metabolites were associated with *Candidatus*_*Saccharimonas* and *Lactobacillus* at the genus level. We analyzed correlations between differential metabolites and the top 15 bacterial genera. Results showed that *Lachnospiraceae*_NK4A136_group, *Bacteroides*, unclassified_f__*Lachnospiraceae*, norank_f__*Muribaculaceae*, and Alistipes had strong correlations with most metabolites. Live K56 and its postbiotic components may influence these gut microbiota, thereby affecting bile acid metabolism, inflammation regulation, and other related pathways to modulate intestinal homeostasis and physiological functions.

## 4. Discussion

Functional constipation remains a globally pervasive gastrointestinal disorder that profoundly compromises patient quality of life [24]. While probiotic-mediated remodeling of the gut microbiota has emerged as a viable therapeutic paradigm to mitigate these clinical symptoms [25], the comparative functional contribution of viable versus non-viable microbial matrices remains inadequately defined. The current research focused on the potential benefits of live and heat-killed K56 in treating constipation induced by loperamide in mice. Our results demonstrated that both forms of K56 effectively shortened the whole gut transit time, increased fecal pellet counts and moisture content, and enhanced the GI transit rate. Notably, live K56 exhibited superior efficacy compared to its heat-killed counterpart in improving these physiological parameters.

The underlying mechanisms by which live and heat-killed K56 alleviate constipation may involve the structural restoration of the colonic muscularis and goblet cells, both of which are key to water uptake and the movement of the intestines [23,26]. According to Shen et al., *L. plantarum* T34 was able to alleviate constipation caused by loperamide in mice by enhancing the thickness of the colonic muscularis [27]. Consistently, a study by Ma et al. regarding the effects of *Lacticaseibacillus rhamnosus* Glory LG12 on constipation also observed a significant thickening of the muscularis propria in the colon [28]. The capacity of both live and heat-killed K56 to increase fecal water content may be attributed to their significant downregulatory effects on the expression of Aqp2 and Aqp3 in colonic tissues [29]. When aquaporins (AQPs) are expressed abnormally in the colon, it can cause excessive water reabsorption and decreased intestinal secretion, resulting in dry stools that obstruct normal defecation [30,31]. Our results align with Zhang et al.’s research, which discovered that kiwifruit polysaccharide and polyphenol extracts reduced colonic Aqp2 and Aqp3 levels during constipation relief [32]. Furthermore, another study indicated that wheat peptides alleviated constipation while simultaneously downregulating the expression of Aqp3 and Aqp4 [33]. Collectively, these data indicate that the live and heat-killed K56 are sufficient to trigger both colon tissue repair and the transcriptional repression of colonic water channels.

Additionally, the alleviation of constipation is tightly connected to neurotransmitters controlling intestinal motility. Regarding neurotransmitters modulation, both live and heat-killed K56 significantly upregulated the levels of pro-kinetic neurotransmitters, namely 5-HT and SP, while concurrently downregulating the expression of the inhibitory neurotransmitter, VIP. Previous studies have established that 5-HT, synthesized and released by enterochromaffin cells, activates enteric nervous reflex arcs to stimulate intestinal smooth muscle contraction and secretory activity [34,35]. Similarly, SP acts as a pro-kinetic neuropeptide that enhances the contractile intensity of intestinal smooth muscles, thereby accelerating the propulsion of intestinal contents [35,36]. In contrast, VIP serves as an inhibitory neurotransmitter that prolongs intestinal transit time by promoting smooth muscle relaxation and suppressing propulsive motility [37]. Under constipated conditions, the elevated expression of Aqp2 and Aqp3 in the colon leads to excessive water reabsorption and diminished intestinal secretion, resulting in reduced fecal moisture and impaired gut motility [31]. These findings suggest that K56 may alleviate constipation by modulating the “5-HT/SP-VIP-AQP” regulatory axis, thereby restoring both smooth muscle motility and intestinal water transport.

Constipation pathogenesis is significantly influenced by gut microbiota dysbiosis. Microbiome analysis in the present study revealed that live K56 significantly increased the abundance of *Lactobacillus* and *Lachnospiraceae*, while effectively curbing the proliferation of potential pathogens, including *Bacteroides*, *Muribaculaceae*, and *Candidatus*_*Saccharimonas*. In contrast, heat-killed K56 primarily exhibited a significant stimulatory effect on the abundance of *Lachnospiraceae*. Both live and heat-killed K56 contributed to the production of SCFAs, such as butyric, acetic, and isobutyric acids, alongside the reconstitution of the microbial structure. Notably, butyric acid—as the primary energy source fueling colonic epithelial cells—plays a multifaceted role in stimulating mucus secretion [38], modulating enteric nervous signaling [39], and exerting potent anti-inflammatory and barrier-repairing effects [40].

Metabolomics analysis further revealed that both live and heat-killed K56 significantly modulated amino acid metabolic pathways, particularly those involving tryptophan metabolism. Tryptophan serves as a critical precursor for 5-HT synthesis, and its metabolic dysregulation is closely linked to enteric nervous system dysfunction in constipation [41]. Studies have indicated that constipated patients often exhibit decreased colonic mucosal TPH1 expression and reduced 5-HT synthesis, whereas SCFAs like butyrate can stimulate 5-HT production [40,42]. In the present study, both live and heat-killed K56 significantly elevated the levels of tryptophan and butyrate, suggesting that they may enhance intestinal peristalsis by activating the tryptophan–5-HT signaling pathway. However, K56-L produced a broader metabolic response, with prominent changes in arginine and proline metabolism, arginine biosynthesis, tryptophan metabolism, and bile acid-related pathways. The levels of L-arginine, spermidine, and other polyamine-related metabolites were increased after K56-L treatment, accompanied by increases in tryptophan-derived metabolites, including tryptamine and 5-HIAA. These changes suggest that K56-L may influence arginine/polyamine and tryptophan metabolism, both of which are involved in intestinal motility and mucosal homeostasis [43]. Several bile acid-related metabolites, including glycocholic acid and dehydrocholic acid, were also increased in the K56-L group, indicating a change in bile acid metabolism. Alterations in fecal bile acid metabolism have been reported in patients with slow-transit constipation, and recent studies have highlighted the role of microbiota-derived bile acids in regulating gastrointestinal motility through bile acid receptors such as TGR5 [44]. In contrast, K56-D caused fewer metabolic changes, mainly involving phenylalanine- and tyrosine-related metabolites, methionine metabolism, and selected bile acid and polyamine metabolites. The increase in N8-acetylspermidine may nevertheless indicate some alteration in polyamine metabolism, which has been implicated in intestinal epithelial renewal and barrier maintenance [45]. Direct comparison of K56-L and K56-D showed higher levels of several bile acid-related metabolites, 5-HIAA, and L-arginine in the K56-L group. Taken together, both preparations affected the fecal metabolic profile, but their responses differed in both the range and magnitude of metabolic changes, with K56-L showing more evident effects on arginine/polyamine, tryptophan, and bile acid metabolism.

Overall, both K56-L and K56-D effectively alleviated constipation, with differences in their efficacy summarized in Table 1.

Finally, some limitations of this study should be considered. The experiments were performed in a loperamide-induced constipation mouse model, and the results may not fully reflect the response in patients with functional constipation. The treatment lasted only 21 days, which did not allow us to assess the long-term efficacy or safety of K56-L and K56-D.

## 5. Conclusions

In conclusion, the findings of this study suggest that both live and heat-killed *L. paracasei* K56 can successfully mitigate constipation in mice caused by loperamide. The underlying mechanisms are primarily attributed to the structural restoration of the colonic muscularis and goblet cells, coupled with the normalization of water transport via the downregulation of colonic Aqp2 and Aqp3. Furthermore, K56 orchestrates the balance between pro-kinetic (5-HT/SP) and inhibitory (VIP) neurotransmitters, enhancing intestinal motility through the “5-HT/SP-VIP-AQP” regulatory axis. Multi-omics analysis further reveals that K56 intervention reshapes the gut microbiota, enriches the abundance of *Lactobacillus* and *Lachnospiraceae*, and boosts the production of SCFA (particularly butyrate), thereby activating the “tryptophan−5-HT” metabolic pathway. Notably, K56-L produced more pronounced changes in metabolic pathways related to arginine/polyamine, tryptophan, and bile acid metabolism, which may contribute to the regulation of intestinal motility and mucosal homeostasis. In contrast, K56-D showed a more limited metabolic response, mainly involving phenylalanine/tyrosine and methionine metabolism. Overall, the research provides a firm mechanistic justification for the use of *L. paracasei* K56, in live and postbiotic forms, as a functional food ingredient aimed at managing constipation.

## Figures and Tables

**Table 1:** Comparison of the main differences between K56-L and K56-D in mice with loperamide-induced constipation.

Parameter	K56-L	K56-D	Main Difference
whole gut transit time	↓	↓	K56-L showed greater improvement
fecal water content	↑	↑	K56-L showed greater improvement
6 h fecal pellet count	↑	↑	K56-L showed greater improvement
small intestinal propulsion rate	↑	↑	K56-L showed greater improvement
butyrate	↑	↑	Greater response with K56-L
Colonic muscularis	Restored	Restored	Both improved
Goblet cells	Restored	Restored	Both improved
AQP2/AQP3	↓	↓	Both treatments reduced expression
*Lactobacillus*	↑	Less pronounced	More evident with K56-L
*Lachnospiraceae*	↑	Less pronounced	More evident with K56-L
Arginine/polyamine metabolism	More pronounced	Limited	K56-L predominant
Tryptophan metabolism	More pronounced	Limited	K56-L predominant
Bile acid metabolism	More pronounced	Some changes	K56-L predominant
Phenylalanine/tyrosine metabolism	—	More evident	K56-D predominant
Methionine-related metabolism	—	More evident	K56-D predominant

↑: significantly increased compared with the Model group; ↓: significantly decreased compared with the Model group; —: no significant change compared with the Model group.

## Data Availability

The original contributions presented in this study are included in the article. Further inquiries can be directed to the corresponding author.

## Supplementary Materials

The following supporting information can be downloaded at https://www.mdpi.com/article/10.3390/nu18182991/s1. Figure S1: K56 and its postbiotics modulate the gut microbiota composition in constipated mice. (A) Relative abundance of gut microbiota at the phylum level; (B) Relative abundance at the family level.; Figure S2: Volcano plot of colon metabolites in constipated mice after Intervention with K56 and its postbiotics intervention. (A) Control VS Model; (B) Model VS K56-L; (C) Model VS K56-D. Figure S3: OPLS-DA Model of Metabolites in the Colon of Constipated Mice After Intervention with K56 and its postbiotics. (A) Control VS Model; (B) Model VS K56-L; (C) Model VS K56-D. Figure S4: OPLS-DA Model fit of Metabolites in the Colon of Constipated Mice After Intervention with K56 and its postbiotics. (A) Control VS Model; (B) Model VS K56-L; (C) Model VS K56-D. Figure S5: Cluster heatmap of differential metabolites. Figure S6: α−diversity analysis − Chao1 index. Figure S7: Melt Curve; Table S1: Primer sequences for qRT-PCR.

## Abbreviations

The following abbreviations are used in this manuscript:

FC	Functional constipation
K56-L	Live K56 group
K56-D	Heat-killed K56 group
VIP	vasoactive intestinal peptide
SP	substance P
5-HT	5-hydroxytryptamine
Aqp2	Aquaporins 2
Aqp3	Aquaporins 3
IL-6	Interleukin-6
TNF-α	Tumor Necrosis Factor-alpha
SCFAs	Short-Chain Fatty Acids
LOP	loperamide
PEG 4000	Polyethylene Glycol 4000 powder
FWC	fecal water content
ASV	Amplicon Sequence Variants
PCoA	Principal Coordinate Analysis
PLS-DA	Partial Least Squares Discrimination Analysis
OPLS-DA	Orthogonal Partial Least Squares Discriminant Analysis
ELISA	Enzyme-Linked ImmunoSorbent Assay
VIP scores	Variable Importance in Projection
5-HIAA	5-hydroxyindole-3-acetic acid
