# Supplementary material for: Comparative Analysis of Live and Heat-Killed Lacticaseibacillus paracasei K56 in Alleviating Functional Constipation
Source: Nutrients. 2026 Sep 12;18(18):2991. doi: 10.3390/nu18182991 (PMC13610784; doi:10.3390/nu18182991)
Supplement: Supplementary file 1 [file nutrients-18-02991-s001.zip › nutrients-4486531-supplementary.pdf]

Supplement

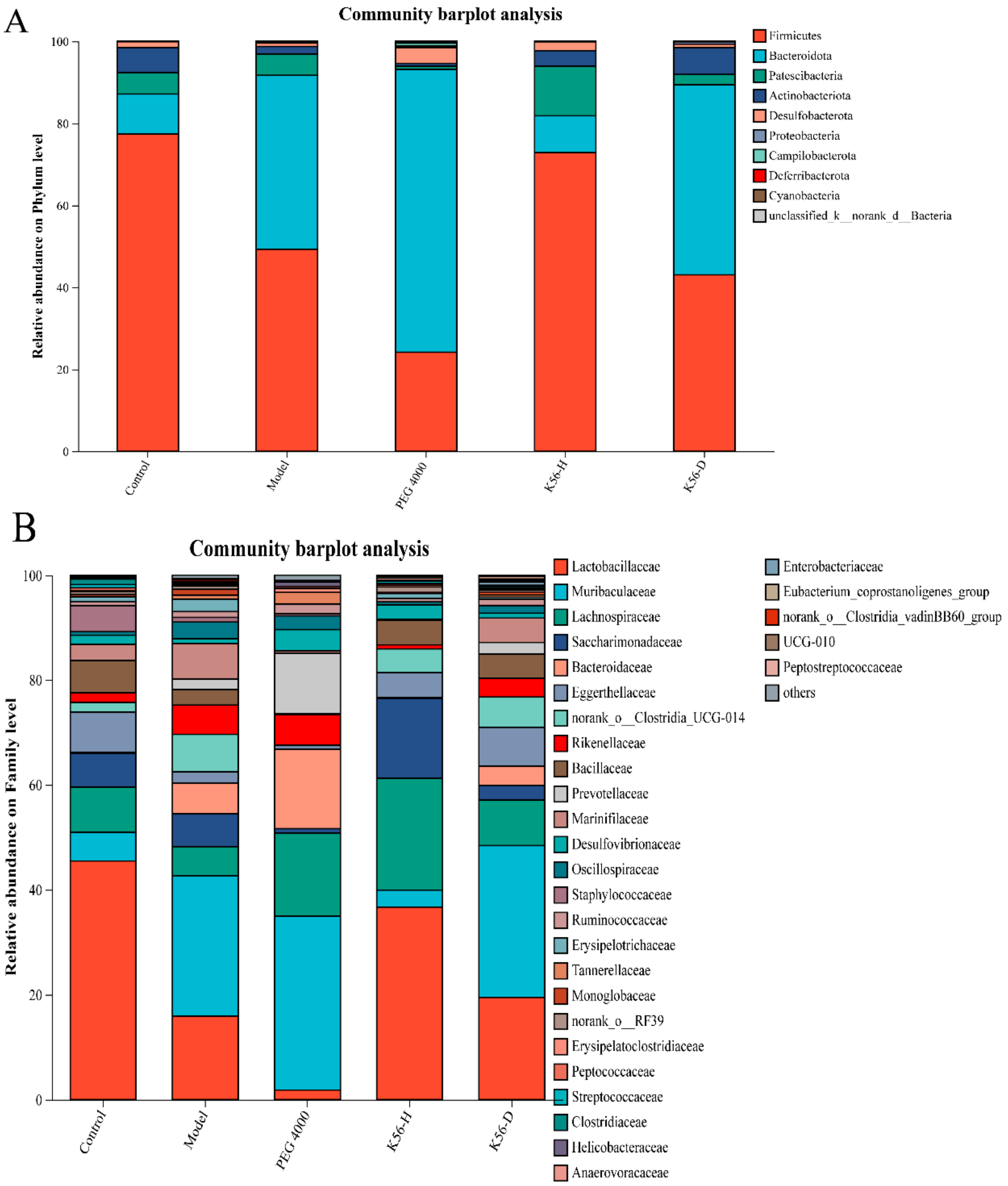

Figure S1. K56 and its postbiotics modulate the gut microbiota composition in constipated mice. (A) Relative abundance of gut microbiota at the phylum level; (B) Relative abundance at the family level.

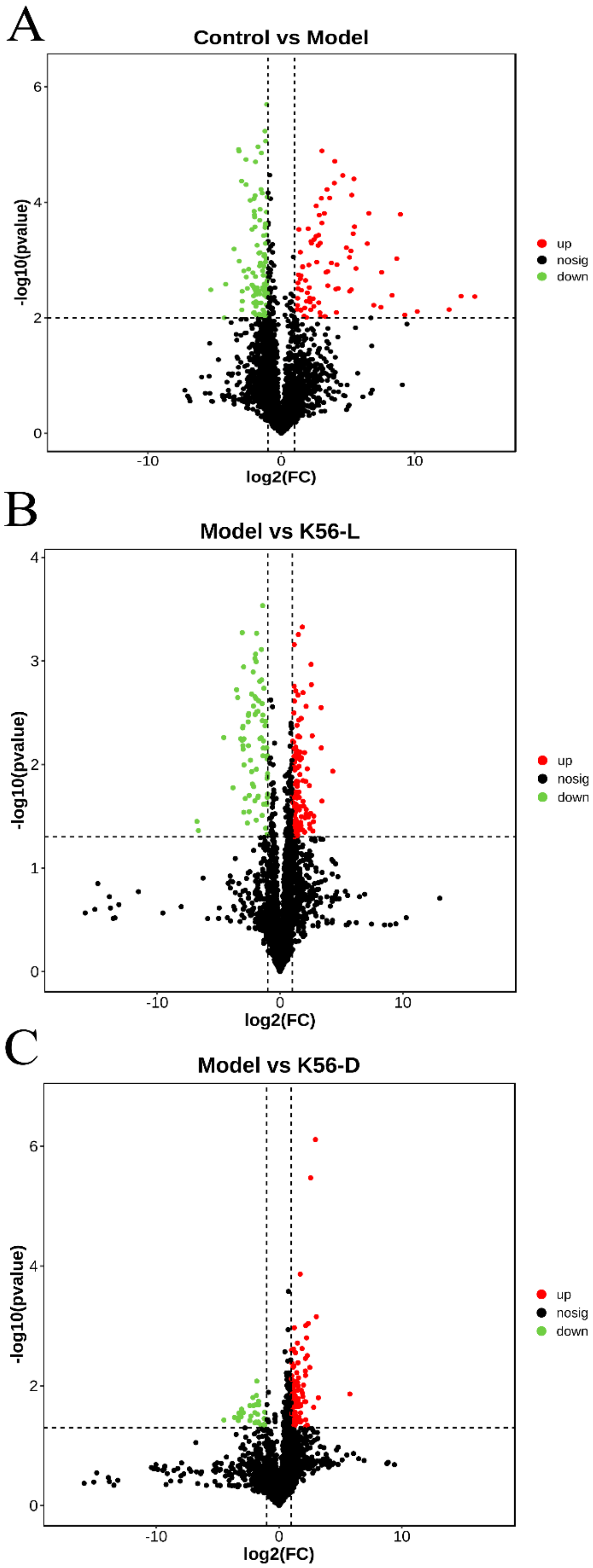

Figure S2. Volcano plot of colon metabolites in constipated mice after Intervention with K56 and its postbiotics intervention. (A) Control VS Model; (B) Model VS K56-L; (C) Model VS K56-D.

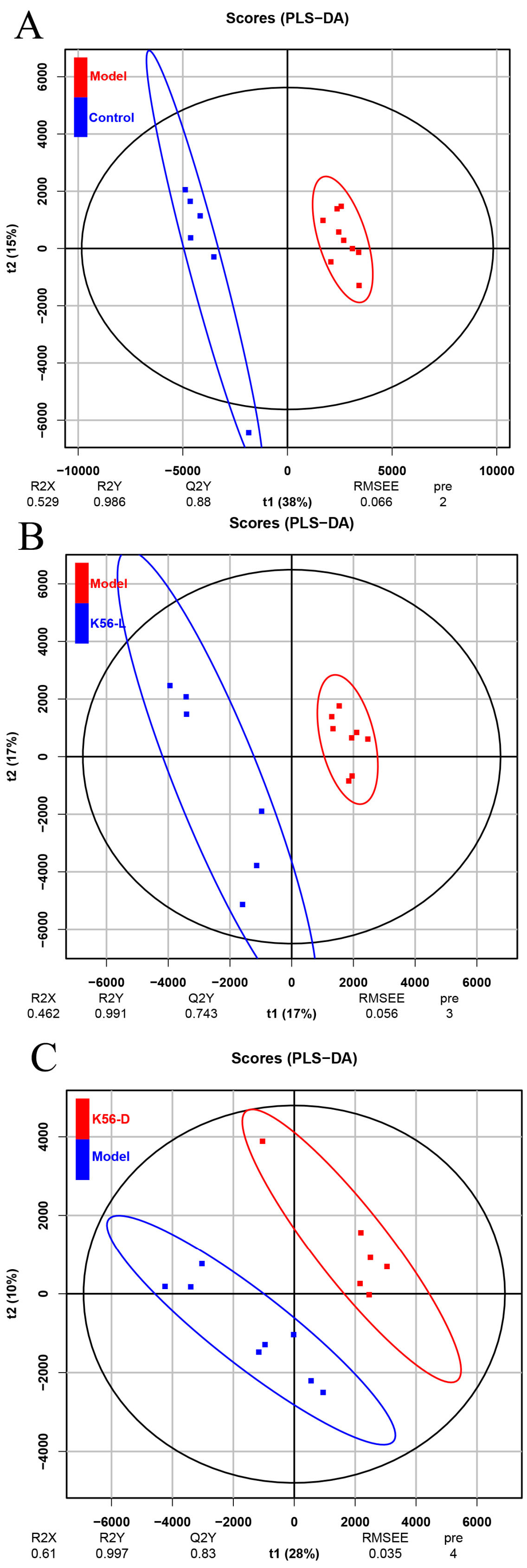

Figure S3. OPLS-DA Model of Metabolites in the Colon of Constipated Mice After Intervention with K56 and its postbiotics. (A) Control VS Model; (B) Model VS K56-L; (C) Model VS K56-D.

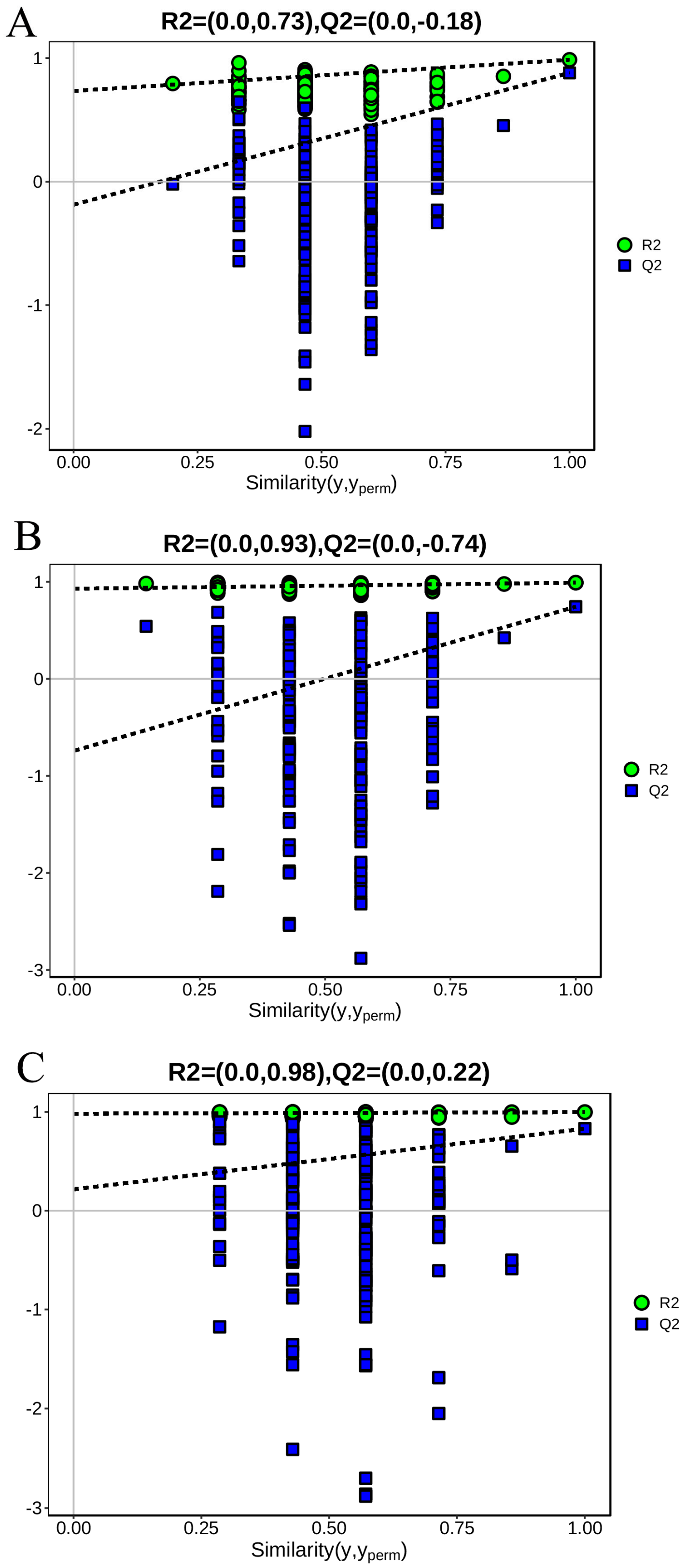

Figure S4. OPLS-DA Model fit of Metabolites in the Colon of Constipated Mice After Intervention with K56 and its postbiotics. (A) Control VS Model; (B) Model VS K56-L; (C) Model VS K56-D.

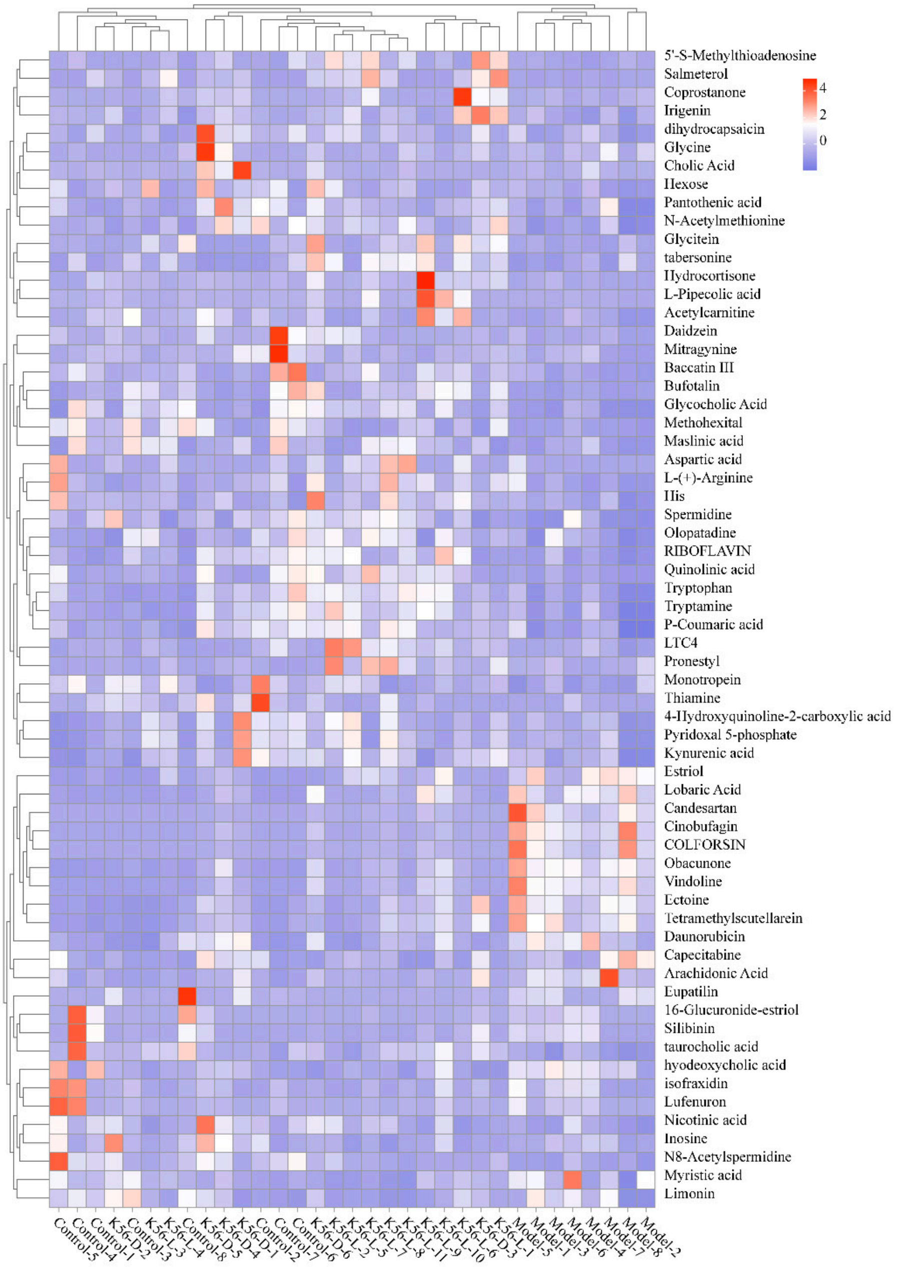

Figure S5. Cluster heatmap of differential metabolites

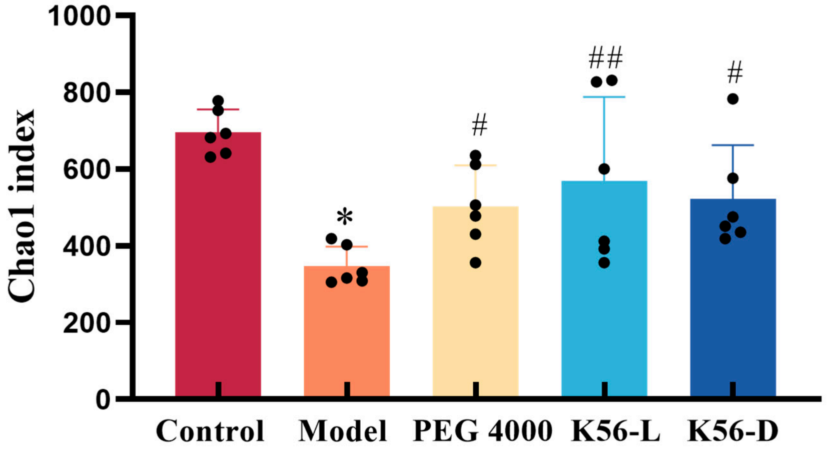

Figure S6. α-diversity analysis - Chao1 index

Table S1. Primer sequences for qRT-PCR

Target Genes	Primer Sense (5′-3′)	Primer Antisense (5′-3′)
AQP2	GGAAGAGCTCCACAGTCACC	CCTCCATGAGATTACCCCTG
AQP3	AAGCCAAGTTGATGGTGAGG	GGGGACCCTCATCCTTGT
IL-6	CCGGAGAGGAGACTTCAC	TCCACGATTTCCAGAGAGA
TNF-α	GGGTGTTCATCCATTCTC	GGAAAGCCCATTTGAGT
GAPDH	ATGGTGAAGGTCGGTGTGAA	TGGAAGATGGTGATGGGCTT

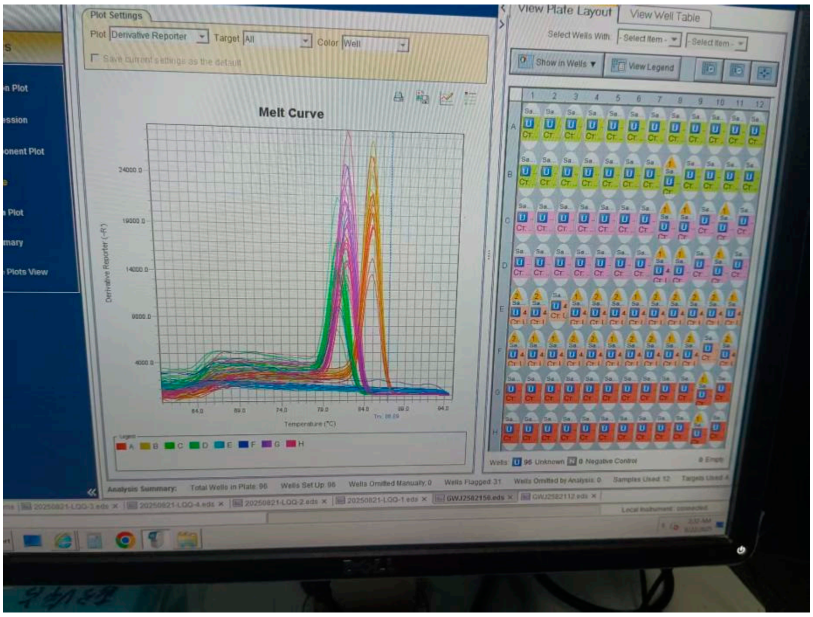

Figure S7. Melt Curve
